# A Case of Effective Balloon Fenestration for Localized Aortic Dissection Complicated with Acute Renal and Bilateral Limb Ischemia in a Thoracoabdominal Aortic Aneurysm

**DOI:** 10.3400/avd.cr.21-00023

**Published:** 2021-06-25

**Authors:** Yusuke Imaeda, Hiroyuki Ishibashi, Yuki Orimoto, Yuki Maruyama, Hiroki Mitsuoka, Takahiro Arima, Ako Isogai, Katsuhiko Matsuyama, Kayo Sugiyama, Kojiro Suzuki

**Affiliations:** 1 Department of Vascular Surgery, Aichi Medical University, Nagakute, Aichi, Japan; 2 Cardiac Surgery, Aichi Medical University, Nagakute, Aichi, Japan; 3 Radiology, Aichi Medical University, Nagakute, Aichi, Japan

**Keywords:** aortic dissection, malperfusion, transcatheter balloon fenestration

## Abstract

An 85-year-old man visited our hospital with bilateral leg weakness. Blood tests revealed an abrupt deterioration of renal function. Computed tomography revealed a 53-mm aortic aneurysm at the level of the diaphragm with an aortic dissection after branching of the superior mesenteric artery. An emergency left axillary–left femoral artery bypass surgery was performed to secure blood flow to the kidneys and lower limbs. Five days later, a transcatheter balloon fenestration for the stenosis was performed, and the blood pressure of the infrarenal aorta was improved. Both the dorsal pedis and posterior tibial arteries became palpable, and renal function was improved.

## Introduction

Emergency surgery is required to treat acute type B aortic dissection complicated by malperfusion. In a frail patient with visceral artery malperfusion due to dissection and anatomically unsuitable for stent graft surgery, endovascular fenestration is occasionally indicated. Herein, we report the case of a thoracoabdominal aortic aneurysm complicated by a localized aortic intimal flap causing bilateral kidney and lower limb ischemia, successfully treated with balloon fenestration.

## Case Report

**Figure 1:**
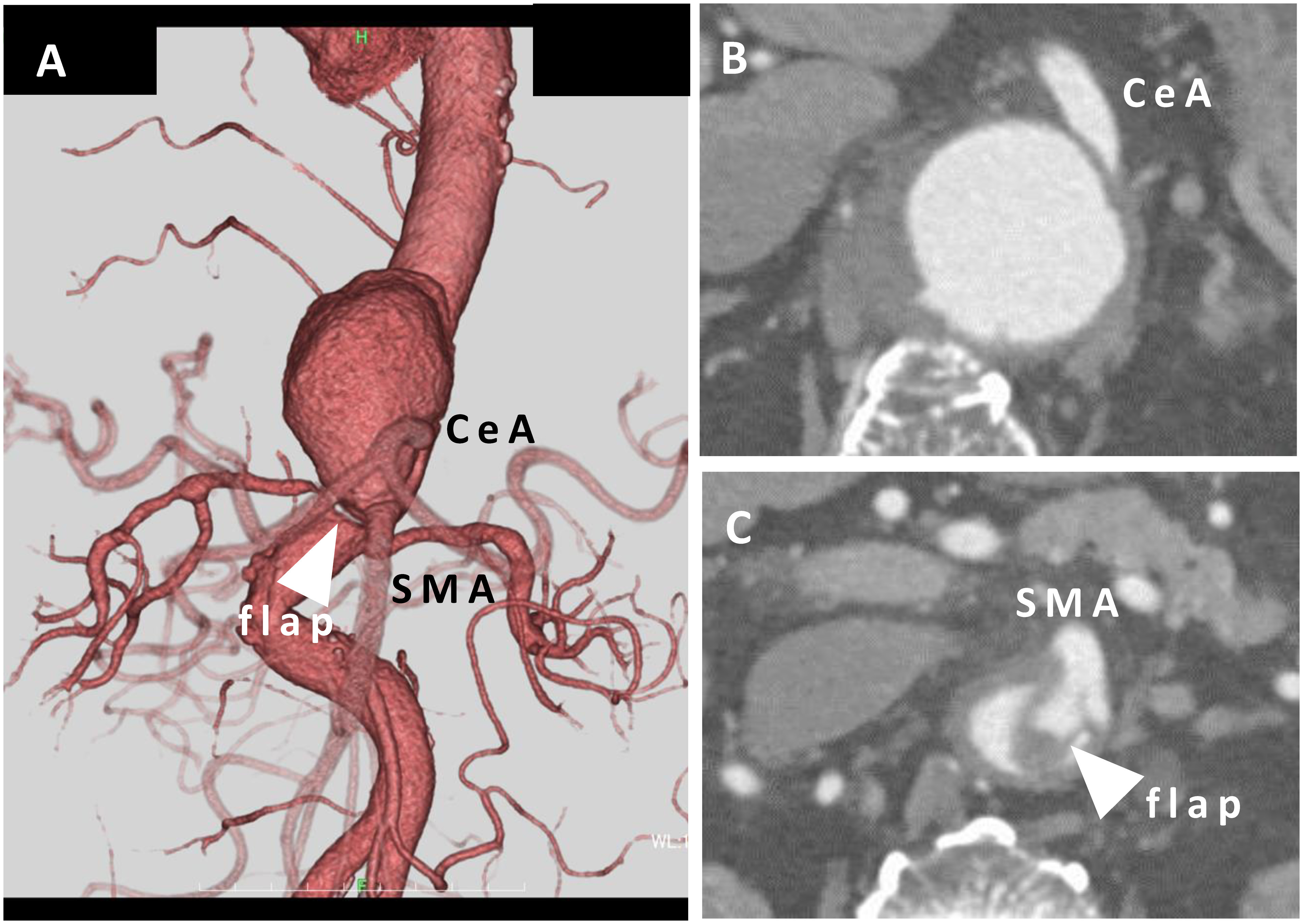
Fig. 1 Contrast-enhanced CT scan. (**A**) 3 dimension CT angiography. There existed an intimal flap due to localized dissection between the SMA and bilateral RAs. (**B**) Celiac artery branched from the 53 mm thoracoabdominal aortic aneurysm. (**C**) Dissection flap was observed at a level of an orifice of the superior mesenteric artery. An entry tear was not detected.

CT: computed tomography; CeA: celiac artery; SMA: superior mesenteric artery; RA: renal artery

An 85-year-old man was transferred to our hospital with bilateral leg weakness. Head computed tomography (CT) and magnetic resonance imaging revealed no abnormalities. Thus, the patient was placed on follow-up observation. His symptoms did not improve, and on the following day, he returned for another consultation. His arterial pulse at both femoral and lower extremity could not be palpated, and leg muscle weakness was evaluated at level 4 of the manual muscle test. Blood examination revealed that renal function had rapidly deteriorated from the previous day (blood urea nitrogen, 7.3–14.5 mg/dL; serum creatinine, 0.94–3.04 mg/dL; and estimated glomerular filtration rate, 58–16 mL/min/1.73 m^2^). Contrast-enhanced CT revealed a 53-mm thoracoabdominal aortic aneurysm at the level of the thoracic diaphragm, and the celiac artery had branched off from the aneurysm (**Figs. 1A** and **1B**). Localized aortic dissection was observed at the level of the superior mesenteric artery, with the true lumen divided and entry not clearly visible (**Fig. 1C**). The blood flow of the bilateral renal and common iliac arteries was decreased, and the contrast effect was delayed in the kidneys. Hence, the patient was diagnosed with malperfusion of both kidneys and legs due to localized aortic dissection with a thoracoabdominal aortic aneurysm. Left axillary–left femoral artery bypass (10-mm J graft®, Japan Lifeline, Tokyo, Japan) was performed on the same day to ensure renal and leg blood flow. After surgery, the urine volume increased (10 mL/h to at least 100 mL/h), and the strength of both legs improved. Because of his high age, transcatheter balloon fenestration of the flap was performed on day 5 after the bypass to ensure bilateral renal and leg blood flow instead of open surgical replacement.

**Figure 2:**
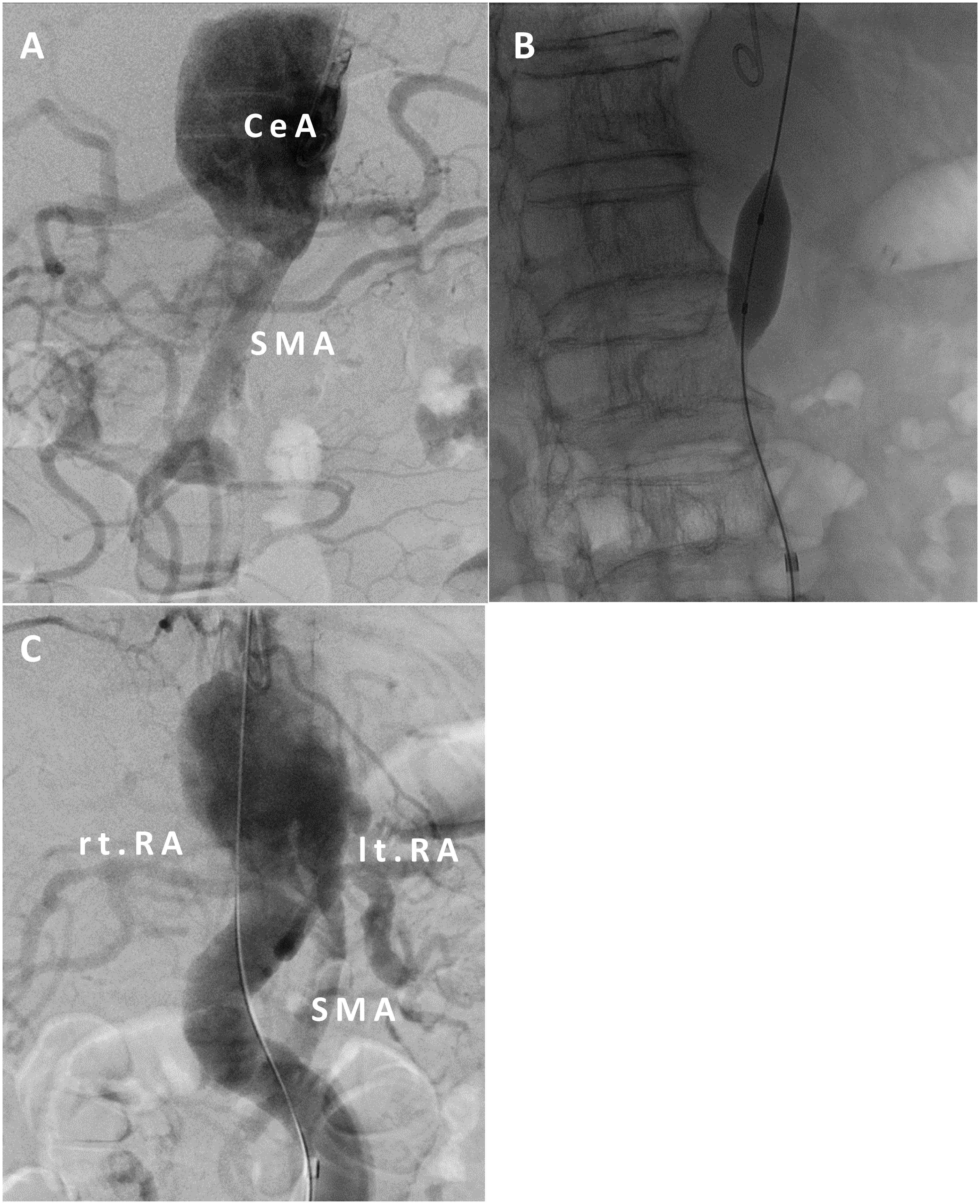
Fig. 2 Digital subtraction angiography of a fenestration. (**A**) Pre-balloon dilation. The abdominal aorta was almost occluded just distal to the SMA. (**B**) Dilation by a 16-mm balloon. (**C**) Post-balloon dilation. The infrarenal aorta and the bilateral renal arteries were well visualized.

CeA: celiac artery; SMA: superior mesenteric artery; lt. RA: left renal artery; rt. RA: right renal artery

**Figure 3:**
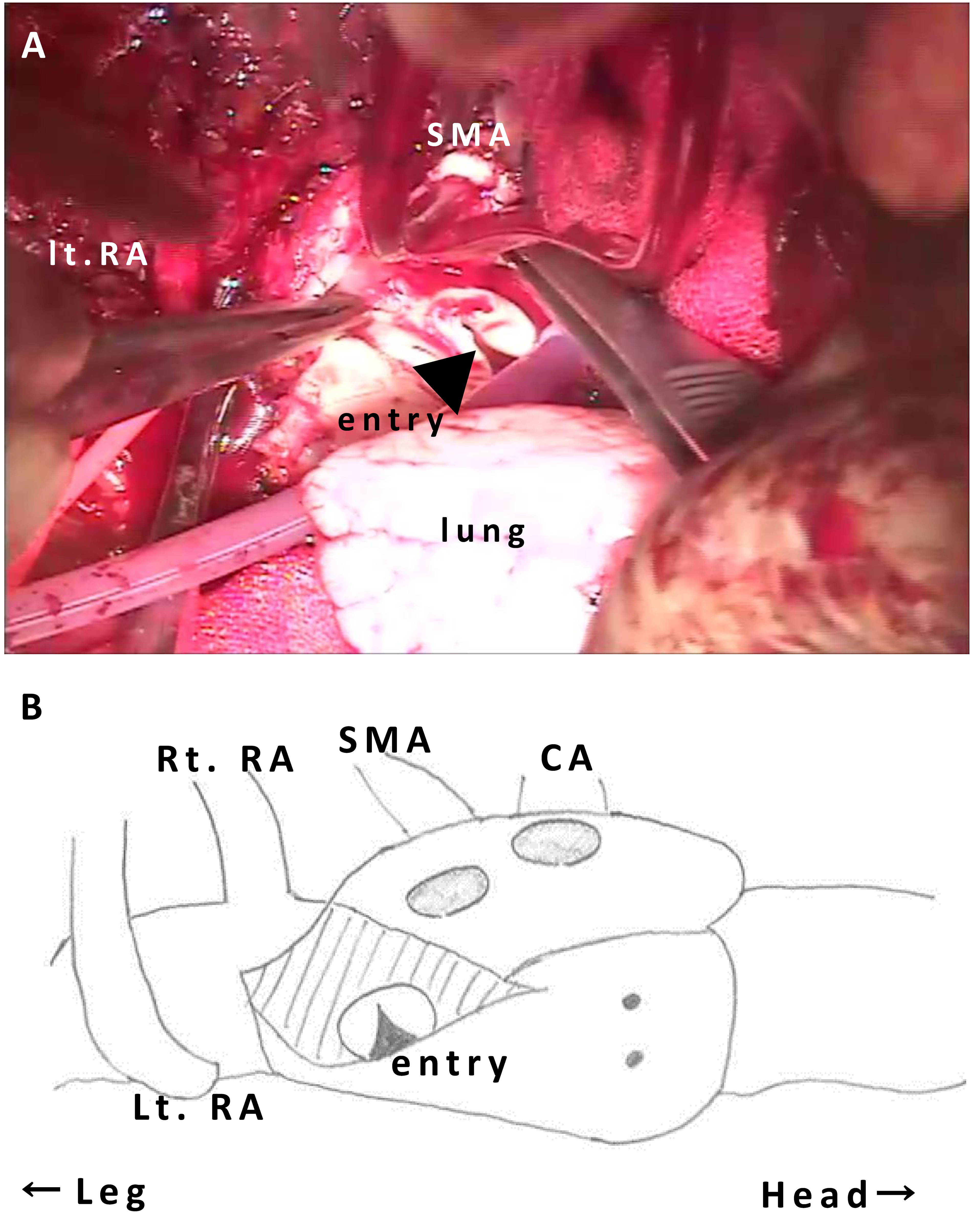
Fig. 3 (**A**) Operative photo of the thoracoabdominal aortic aneurysm surgery. An entry created via fenestration was recognized in the center of the photo. (**B**) The schema of the thoracoabdominal aortic aneurysm surgery.

Fenestration was performed under general anesthesia. A pigtail catheter was inserted from the left brachial artery, and abdominal aortography was performed. It showed occlusion of the abdominal aorta just distal to the superior mesenteric artery (**Fig. 2A**). A 7-Fr sheath was inserted from the right common femoral artery, and a 0.035-inch Radifocus® (TERMO, Tokyo, Japan) guidewire was successfully passed through a narrow channel of the dissection to the descending thoracic aorta. Following systemic administration of 3000 units of heparin, the entry was expanded with an 8-mm Mustang® PTA balloon (Boston Scientific Corporation, Marlborough, MA, USA), followed by a 16-mm Gekira® PTA balloon (COSMOTEC, Tokyo, Japan) (**Fig. 2B**). Subsequent to the balloon fenestration, the aortic blood pressure distal to the stenosis improved from 62 mmHg to 86 mmHg, and the pressure gradient decreased from 46 mmHg to 23 mmHg. Finally, the infrarenal aorta and bilateral renal arteries were well visualized (**Fig. 2C**). Moreover, the patient’s pulse could be palpated on the dorsalis pedis and posterior tibial artery. The ankle–brachial blood pressure index was 0.64 on the right and 0.75 on the left. Renal function also recovered, with serum creatinine levels at 0.79 mg/dL. He was discharged on day 22 after the fenestration. On his first outpatient visit at 1 month after surgery, CT revealed rapid enlargement of the aneurysm by 5 mm, reaching 60 mm in diameter. Therefore, graft replacement surgery was performed. Operative findings showed an intimal flap inside the aorta at the level of the superior mesenteric artery, and an approximately 10 mm balloon-fenestrated entry was observed (**Fig. 3**). He was discharged 12 days after surgery. He is currently in good health and attends outpatient visits.

CA: celiac artery; Lt. RA: left renal artery; Rt. RA: right renal artery; SMA: superior mesenteric artery

## Discussion

Malperfusion due to aortic dissection has an incident rate of 18%–33%.^1)^ For aortic dissection complicated with malperfusion, open surgical repair or stent graft surgery is indicated.^2)^ Although stent graft surgery has become a mainstay in type B aortic dissection in recent years, some cases are difficult because of anatomical reasons. In the present case, the entry was located close to the major abdominal tributaries. In addition, open surgical repair using extracorporeal circulation was risky because of his advanced age and comorbidity; thus, transcatheter fenestration became an option to treat malperfusion for this case. In transcatheter fenestration, an entry of the dissection is expanded with a balloon catheter to increase blood flow to distal organs. Another report revealed placement of a stent at a fenestrated entry.^3)^ One advantage of the fenestration technique is that it is less invasive and potentially improves blood flow in less time.^4)^ However, it has some disadvantages: it cannot be performed if the guidewire cannot be passed through the narrow entry.^5)^ If the narrow entry is not passed, a puncture device, including a transjugular intrahepatic portosystemic shunt needle or Brockenbrough needle is necessary.^5)^ With rapidly progressing renal deterioration and lower limb ischemia, the present case was considered to require rapid revascularization. However, the location of the entry tear was unclear on contrast-enhanced CT scan; thus, emergency axillary–femoral artery bypass was performed to secure blood flow to the lower body, and elective balloon fenestration was planned thereafter. Although it was not difficult to pass the guidewire through the narrow entry in the present case, it was thought that several puncture devices and intravascular ultrasonography were required for this procedure.

It has been reported that true aortic aneurysms complicate aortic dissection at a frequency of approximately 5%–7%,^6,7)^ and there are some case reports in the Japanese literature.^8,9)^ However, to the best of our knowledge, it is quite rare that the localized dissection occurred at the end of a thoracoabdominal aortic aneurysm and was complicated with organ malperfusion. In the present case, there was no pain preceding the dissection, and the onset of the dissection was unknown. The main symptom was ischemia of the lower body. This case was considered uncommon on the basis of the aforementioned points. Although the aneurysm was enlarged after fenestration, requiring graft replacement, it is unclear whether balloon fenestration affects the hemodynamics in the vicinity of the aneurysm. However, there are reports of increased false lumen blood flow due to balloon fenestration, resulting in aneurysm formation with the enlargement of the false lumen.^4,10)^ Thus, careful follow-up is important after balloon fenestration if this procedure has been performed for aortic aneurysm complicated with aortic dissection, as in the present case.

## Conclusion

This case is an example of effective balloon fenestration for localized aortic dissection complicated with acute renal and bilateral limb ischemia in a thoracoabdominal aortic aneurysm.
